# The transcription factor *SKN7* regulates conidiation, thermotolerance, apoptotic-like cell death and parasitism in the nematode endoparasitic fungus *Hirsutella minnesotensis*

**DOI:** 10.1038/srep30047

**Published:** 2016-07-20

**Authors:** Muzammil Hussain, M. Imran Hamid, Niuniu Wang, Lin Bin, Meichun Xiang, Xingzhong Liu

**Affiliations:** 1 State Key Laboratory of Mycology, Institute of Microbiology, Chinese Academy of Sciences, No 3 Park 1, Beichen West Rd., Chaoyang District, Beijing 100101, China; 2 University of Chinese Academy of Sciences, Beijing, China; 3 Department of Plant Pathology, University College of Agriculture, University of Sargodha, Sargodha, 40100, Pakistan

## Abstract

The transcription factor *SKN7* is a highly conserved protein among fungi and was initially recognized as a response regulator that protects cells from oxidative stress and maintains cell wall integrity in yeast. Orthologs of *SKN7* are extensively present in biocontrol agents of plant pathogens, but they had not been functionally characterized. Here, we identified and characterized the transcription factor *SKN7* in the nematode endoparasitic fungus *Hirsutella minnesotensis*. Null mutant lacking *HIM*-*SKN7 (HIM_03620*), which was generated by a gene disruption strategy, demonstrated reduced conidiation, increased sensitivity to high temperature, hydrogen peroxide, mannitol and ethanol, and reduced fungal resistance to farnesol. However, over-expression mutant showed increased conidial production, thermotolerance and resistance to farnesol, suggesting that *HIM-SKN7* regulates antiapoptotic-like cell death in *H. minnesotensis*. Moreover, the results showed that in null mutant, *H. minnesotensis* had decreased endoparasitic ability as compared to wild type and over-expression strain. During the infection process, the relative expression of the *HIM-SKN7* gene was significantly induced in the wild type and over-expression strain. The results of the present study advance our understanding of the functions of the *SKN7* gene in biocontrol agents, in particular, nematode endoparasitic fungi.

*Hirsutella minnesotensis* (Ophiocordycipitaceae, Hypocreales, Ascomycota) is the dominant biocontrol agent of *Heterodera glycines* (soybean cyst nematode, SCN) and is a representative nematode endoparasitic fungi in nature. The species produces globose conidia that adhere to the cuticle during the movement of the secondary stage juvenile (J2) of SCN and subsequently penetrate, digest and eventually kill the nematode^1^,^2^,^3^. Field and greenhouse experiments have shown that *H. minnesotensis* is a potent factor that regulates the SCN population in the soil and contributes to soil suppressiveness in a soybean monoculture system^4^,^5^. Adequate conidial production in soil is important for *H. minnesotensis* to parasitize SCN. Environmental factors, such as temperature, soil type and water content, which are required for the efficient activity of *H. minnesotensis*, have been studied under diverse conditions^6^. Conidia activity may be perturbed by temperature, nutrition, abiotic stresses and secondary metabolites^7^,^8^, but conidial cells are capable of combating high temperature, ultraviolet radiation and the generation of reactive oxygen species intermediates during respiration. Fungi respond to these unfavorable conditions by inducing the expression of stress related proteins, among that the role of heat shock transcription factor and its homolog *SKN7* plays central part.

Heat shock proteins are a conserved family of proteins that are encoded by several heat shock genes. The expression of these genes is regulated by heat shock transcription factor proteins (HSPs). Heat shock transcription factors (HSFs) recognize conserved DNA binding motifs (heat shock elements, HSEs) that are found in the promoter region of heat shock genes, and activate their transcription to promote survival and respond to abiotic stress, temperature and pathogen infection^9^,^10^. There is a high degree of homology between the sequence of *SKN7* and the DNA binding domain of heat shock transcription factors (HSFs). A Skn7 gene contains a potential receiver domain, which is found in the two-component signal transduction family of proteins in prokaryotes and is extensively present across the kingdoms^11^,^12^,^13^. Skn7 is a stress-responsive transcription factor with a uniform architecture that is composed of a N-terminal DNA binding domain that is homologous to a heat shock transcription factor (HSF) and a C-terminal receiver domain^14^. These domains are highly conserved among fungi. The receiver domain regulates Skn7 transcriptional activity by His-Asp phosphorelay signaling via the phosphorylation of a conserved aspartate^15^. As such, Skn7 is an important regulatory factor that regulates multi-functional responses in fungi and other organisms.

In yeast, it has been revealed that *SKN7* and *HSF1* collaborate to achieve the maximal induction of heat shock genes in response to oxidative stress^16^, and the disruption of *SKN7* in *Saccharomyces cerevisiae* resulted in sensitivity to oxidizing agents and the induction of TRX2 genes^17^. The deletion of *SKN7* gene, which is also known as *POS9*^18^ and *BRY1*^19^, resulted in the sensitivity of yeast cells to hydrogen peroxide. The over-expression of *SKN7* inhibited cell wall defects that have been linked to the mutation of *KRE9* gene^14^, as well as the lethality associated with the loss of the G_1_ transcription factors *SBF* and *MBF*, indicating the role of *SKN7* in the regulation of the cell cycle^19^. Moreover, the disruption of *SKN7* was shown to be lethal in *pkc1*∆ background^19^, and the *PKC1* MAP kinase pathway has been reported to be involved in cell wall biosynthesis^20^. In the human fungal pathogen *Cryptococcus neoformans, SKN7* mutants were less virulent and sensitive to reactive oxygen species, as compared to those with wild-type^21^. These results suggest that *SKN7* plays functional roles in the response to different stresses and might be involved in the expression of genes that regulate the cell wall.

*H. minnesotensis* diverged from *Ophiocordyceps sinensis* around 23.9–33.9 Mya and from *Tolypocladium inflatum* around 29.7–39.7 Mya. Phylogenetic studies suggest that *H. minnesotensis* clusters with entomopathogens and is closely linked to the caterpillar fungus *O. sinensis*^2^. When analyzing the function of Skn7 in other fungi, it was of immense interest to investigate the role of Skn7 in *H. minnesotensis*, a dominant endoparasite of *H. glycines*. In the present study, we have functionally characterized a gene within the *H. minnesotensis* wild type strain, WT-3608, encoding a HSF-type DNA-binding domain along with REC (signal receiver domain), and designated it as transcription factor, *HIM-SKN7*. We developed *HIM-SKN7* knockout and over-expression mutant of *H. minnesotensis* WT-3608 and found that *HIM-SKN7* regulates conidiation, thermotolerance, abiotic stress resistance, antiapoptotic like cell death and nematode endoparasitic efficiency.

## Results

### Phylogeny, gene structure and motifs analyses

**Figure 1:**
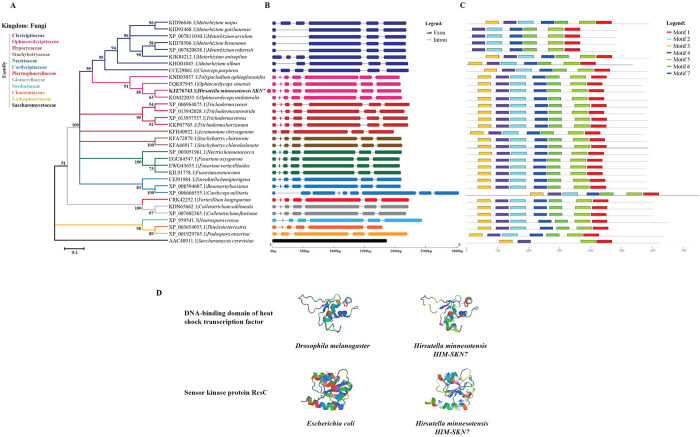
Phylogenetic analysis, gene structure and motifs comparison. (**A**) Phylogenetic analysis of the different orthologs of *HIM-SKN7* (**KJZ76743.1|*****Hirsutella minnesotensis SKN7***). The protein sequences were aligned with ClustalW, and a Maximum likelihood (ML) tree was generated using a WAG model. **(B)** Genomic and coding sequences were used for gene structure analysis to identify the number of introns and exons. **(C)** The motifs analysis comparison showed various conserved regions in the protein sequences of *HIM-SKN7* and its different orthologs. (**D**) Prediction of the tertiary structure of the conserved domain in *HIM-SKN7* and comparison with that of *Drosophila melanogaster* and *Escherichia coli*.

The *HIM-SKN7* ORF sequence was 2105 bp and encoded the HSF-type DNA-binding domain and REC (signal receiver domain). The coding sequence of *HIM-SKN7* was 1737 bp and encoded a protein sequence of 578 amino acids, with an estimated molecular mass of 63 kDa and an isoelectric point of 6.29. The phylogenetic analysis revealed that *HIM-SKN7* (KJZ76743.1) is closely related to the homolog *Ophiocordyceps unilateralis* (KOM22035.1), an entomopathogenic fungus, and therefore is clustered with different families of hypocrealean fungi (Fig. 1A). Gene structure analysis demonstrated that *HIM-SKN7* and its orthologous genes contained 3 to 8 introns. However, most commonly, the genes had 6 introns of different length (Fig. 1B). Analysis of the conserved motifs in *HIM-SKN7* and its orthologs revealed 6–7 motifs, with an average width ranging from 42–50. Motif 5 was not present in the protein sequences of KID92468.1, XP_007811010.1, KID78506.1 and XP_007820838.1 (Fig. 1C). The prediction of the tertiary structure showed 40% similarity between the HSF DNA-binding domain of *HIM-SKN7* and *Drosophila melanogaster*, and 34% similarity with the sensor kinase protein ResC from *Escherichia coli* (Fig. 1D).

### Targeted gene disruption, over-expression and phenotypic characterization

**Figure 2:**
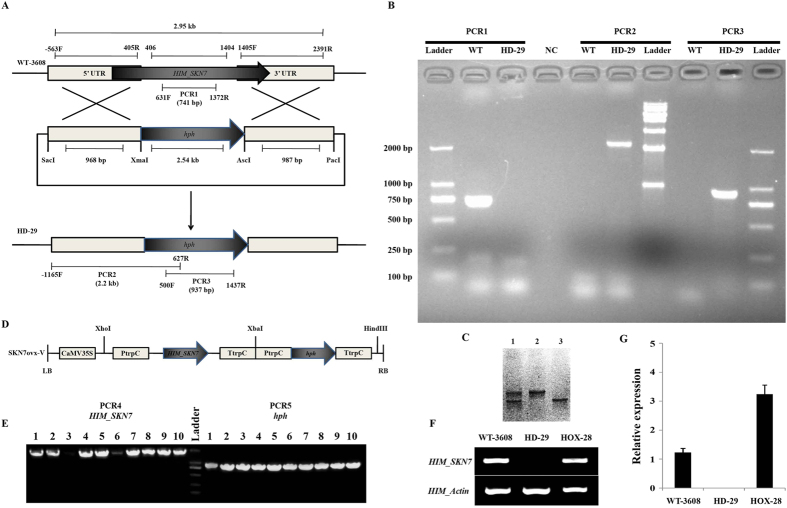
Generation of *HIM-SKN7* disruption and over-expression mutant. (**A**) A schematic illustration for the disruption of the *HIM-SKN7* gene. The *hph* gene was used as a selection marker after transformation. (**B**) PCR verification. PCR1 was performed to amplify the disrupted region of the *HIM-SKN7* gene. The expected product size was 741 kb for WT, and no amplification was expected for knockout strain. PCR2 was performed to verify the replacement of the *HIM-SKN7* gene by the *hph* gene. The forward primer was designed to bind 0.6 kb upstream of the 5′UTR (used in vector for replacement), and the reverse primer designed to bind near the center of the *hph gene*. The expected product size of mutant was 2.2 kb, and no amplification was expected for the WT strain. PCR3 was performed to amplify the *hph* gene from the mutant. The expected product size was 937 bp for the mutant and no amplification was expected for the WT. (**C**) Amplification of *HIM-SKN7* ORF. lane 1, negative transformants; lane 2, positive transformants; lane 3, wild-type 3608. (**D**) A schematic illustration for the over-expression of the *HIM-SKN7* gene. The *hph* gene was used as a selection marker after transformation. (**E**) PCR verification. PCR4 was performed to ensure the correct orientation of the *HIM-SKN7* gene in the vector, pCAMBIA 3300. The forward primer was designed to bind to the promoter region, and reverse primer was designed to bind to the gene. The expected product size was ~1.9 kb. PCR5 was performed to confirm the presence of the *hph* gene in mutants. The expected product size was 937 bp. (**F**) RT-PCR verification to confirm the loss of *HIM-SKN7* transcripts from knockout HD-29. (**G**) Real-time RT-PCR verification to confirm the over-expression of the *HIM-SKN7* gene. The actin gene was used as an internal control.

The functional capabilities of *HIM-SKN7* were investigated in the wild type strain of *H. minnesotensis* (WT-3608) by performing targeted gene disruption and an over-expression strategy (Fig. 2A,D). The gene-disrupted and over-expression transformants were screened phenotypically on potato dextrose agar (PDA) supplemented with hygromycin (*hph*) for revealing the function of *HIM-SKN7*, and the results were confirmed by PCR (Fig. 2B,C,E), RT-PCR (Fig. 2F) and real-time RT-PCR (Fig. 2G). The relative expression analysis showed that the knockout (HD-29) strain showed no transcription activity and the over-expression (HOX-28) strain showed an increase in transcript levels, as compared to WT-3608. The knockout HD-29 showed no significant difference, while over-expression HOX-28 showed a significant increase in mycelial growth, as compared to WT-3608. Moreover, HD-29 produced fewer conidia and fresh biomass, as compared to HOX-28 and WT-3608 (Table 1). These results revealed the prominent role of *HIM-SKN7* in the phenotypic characteristics of the nematode endoparasitic fungus *H. minnesotensis*.

### Deletion of *HIM-SKN7* increased the sensitivity of *H. minnesotensis* to heat shock

**Figure 3:**
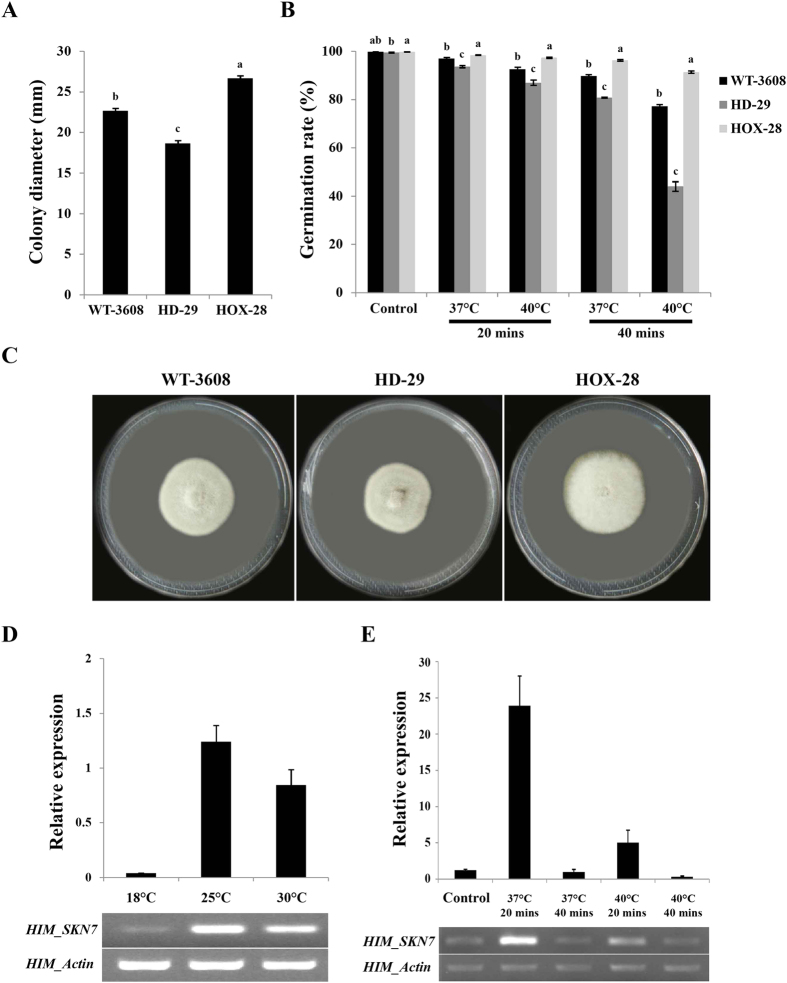
*H. minnesotensis* survival assays under heat stress challenge. (**A**) The colony diameter of *H. minnesotensis* strains that were heat shocked at 37 °C for 2 h. (**B**) The conidial germination percentage of WT-3608, HD-29 and HOX-28 after being exposing to heat shock at 37 °C and 40 °C for 20 and 40 minutes. (**C**) The colony morphology of *H. minnesotensis* WT-3608, HD-29 and HOX-28 at 25 °C after heat shock for 2 h at 37 °C. (**D**) The relative expression of *HIM-SKN7* in WT-3608 at 18 °C, 25 °C and 30 °C. (**E**) The relative expression of *HIM-SKN7* in WT-3608 after heat shock at 37 °C and 40 °C for 20 and 40 minutes along with control. The actin gene was used as an internal control.

To reveal the function of *HIM-SKN7* in response to high temperatures, we first cultured WT-3608 on PDA, and incubated at temperature ranging from 18 °C to 40 °C for 14 days. We found no significant difference in mycelial growth in WT-3608 grown at temperature ranging from 18 °C to 30 °C. Fungal growth was completely suppressed at temperatures ≥37 °C. When we compared the transcript level of WT-3608 grown at 18 °C and 30 °C, the relative expressions were significantly changed, but did not observe any increases with that of control at 25 °C (Fig. 3D). To test the thermotolerance of *H. minnesotensis* against high temperature and time, 5 mm mycelium agar plug from WT-3608, HD-29 and HOX-28 were heat shocked for 2 h at 37 °C and inoculated on fresh PDA plates. Mycelial growth was triggered after 7 days and significantly inhibited in the knock out strain, HD-29, as compared to WT-3608 and the over-expression strain, HOX-28 (Fig. 3A,C). Subsequently, *H. minnesotensis* WT-3608 was challenged with heat shock at 37 °C and 40 °C for 20 and 40 minutes, and the transcript levels were measured following these two high temperature treatments for two different time periods. The expression levels were increased to 20 fold at 37 °C for 20 minutes and started reduces with increased temperature and time (Fig. 3E). To examine the effect of high temperature on conidia germination and survival, the spores of all strains were heat shocked at 37 °C and 40 °C for 20 and 40 minutes along with control. We found a significantly lower germination rate for HD-29, as compared to WT-3608 and HOX-28 (Fig. 3B). These observations indicate that the *HIM-SKN7* gene also regulates thermotolerance, and protects *H. minnesotensis* from adverse temperature conditions.

### *HIM-SKN7* is required to combat abiotic stresses

**Figure 4:**
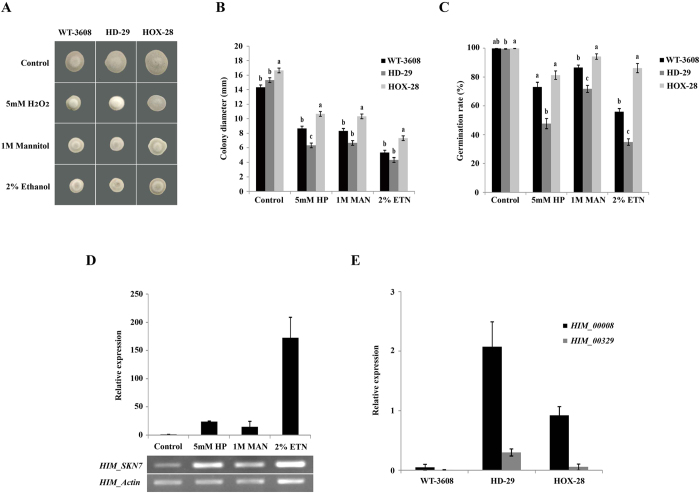
Growth assay and conidia survival assay following abiotic stress challenge. (**A**) The phenotypes of WT-3608, HD-29 and HOX-28 grown on PDA amended with 5 mM H_2_O_2_ (HP), 1 M mannitol (MAN) and 2% ethanol (ETN) for 14 days along with untreated control. (**B**) The colony diameter (mm) of all strains in the presence of 5mM H_2_O_2_ (HP), 1 M mannitol (MAN) and 2% ethanol (ETN) along with untreated control. The data were measured at 14 dpi. (**C**) The spore germination percentage after abiotic stress treatments. All strains were sensitive to 5 mM H_2_O_2_ (HP), 1 M mannitol (MAN) and 2% ethanol (ETN). (**D**) Transcription and relative expression of the *HIM-SKN7* gene in response to 5 mM H_2_O_2_ (HP), 1M mannitol (MAN) and 2% ethanol (ETN) along with control. The WT-3608 were grown in PDB for 4 d and used for RT-PCR and qRT-PCR. The actin gene was used as an internal control. (**E**) The relative expression of heat shock transcription factors, *HIM_00008* and *HIM_00329*, in WT-3608, knockout HD-29 and over-expressed HOX-28.

Abiotic stresses, such as 5 mM H_2_O_2_, 1M mannitol and 2% ethanol, were added to PDA along with untreated control and all strains were cultured for 14 days at 25 °C to compare mycelia growth rate. The knock out HD-29, was sensitive to all stress treatments, as compared to WT-3608, while the over-expression strain, HOX-28, showed rapid mycelial growth under all stresses (Fig. 4A,B). To determine the conidial germination percentage, the conidial suspensions were treated with 5 mM H_2_O_2_, 1 M mannitol and 2% ethanol. Significant differences in the germination rates of all strains were observed, and HOX-28 showed a higher germination percentage than WT-3608 and HD-29. The conidial germination capability of the knockout strain, HD-29, was reduced to 15–25% under different stresses (Fig. 4C). In WT-3608, an increase in the relative expressions levels of *HIM-SKN7* was also observed under these stress conditions (Fig. 4D). The relative expressions levels of heat and stress regulating heat shock transcription factors (HSFs), designated as *HIM_00008* (KJZ80158.1) and *HIM_00329* (KJZ80479.1), were also increased in the knockout strain, HD-29 and over-expression strain, HOX-28 (Fig. 4E). These results showed that *HIM-SKN7* stabilizes cell activity by increasing its expression levels under different stress conditions, and cooperates with heat shock transcription factors in *H. minnesotensis* in order to combat abiotic stresses.

### *HIM-SKN7* is involved in anti-apoptotic like cell death in *H. minnesotensis*

**Figure 5:**
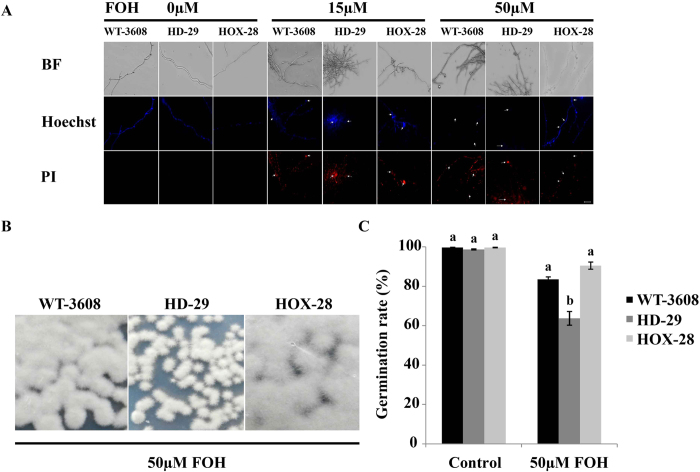
Induction of apoptosis in *H. minnesotensis*. (**A**) 10 days old germlings from WT-3608, HD-29 and HOX-28 were treated with farnesol (FOH) for 4 h at 25 °C, and the samples were double stained with Hoechst 33342/PI dyes. Fluorescence microscopy showed dose dependent apoptosis and necrosis of hyphae at 15 μM and 50 μM FOH. Bar = 50 μm. (**B**) The conidia survival assay on PDA treated with 50 μM FOH. (**C**) The conidia germination percentage of all strains exposed to FOH. HD-29 was more sensitive than WT-3608 and HOX-28 to 50 μM FOH.

To investigate the contribution of *HIM-SKN7* in apoptotic like cell death, we exposed germlings of WT-3608, HD-29 and HOX-28 to the various concentrations (0 μM, 15 μM and 50 μM) of the apoptosis-inducing compound, farnesol (FOH). The FOH-treated mycelium and conidia were co-stained with Hoechst and propidium iodide (PI) dyes and observed under a fluorescence microscope. The microscopy results showed high levels of apoptosis and necrosis-like features, such as intense chromatin condensation and marginalization, in all strains. A dose of 50 μM was observed to be highly toxic and caused severe necrosis in HD-29 and WT-3608 (Fig. 5A). For the conidial survival assay, the conidia of all strains were treated with 50 μM FOH along with blank control. The treatments had significant effect and resulted in decreased conidial germination in the knock out HD-29, as compared to WT-3608 and HOX-28 (Fig. 5B,C). These results indicate that *HIM-SKN7* regulates apoptotic-like cell death and provides functional cell stability in *H. minnesotensis*.

### Disruption of *HIM-SKN7* reduces the nematode endoparasitic ability of *H. minnesotensis*

**Figure 6:**
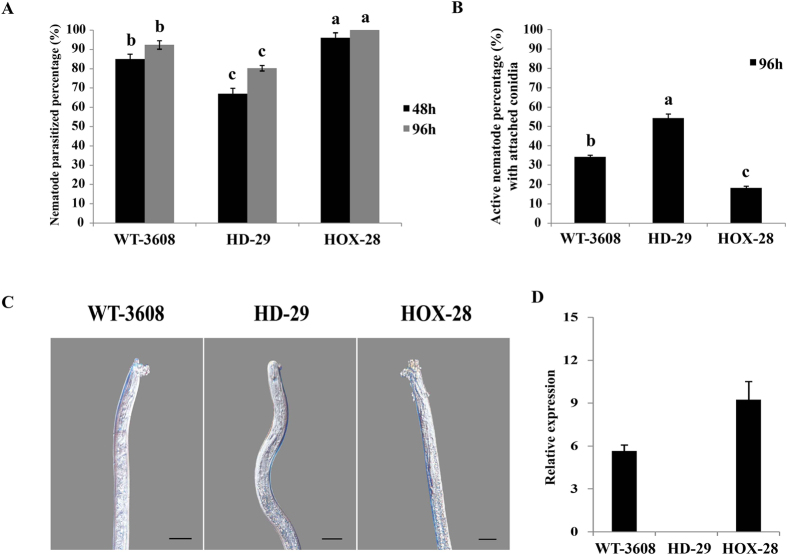
Parasitism of *Heterodera glycines* J2 (SCN) by *Hirsutella minnesotensis*. (**A**) The percentage of nematodes parasitized by WT-3608, HD-29 and HOX-28 at 48 h and 96 h of SCN inoculation. HD-29 parasitized fewer J2, as compared to WT-3608 and HOX-28. (**B**) The percentage of active J2 with adhered conidia. The highest mortality rate was recorded in HOX-28, followed by WT-3608 and HD-29. The data were recorded at 96 h. (**C**) Conidia of *H. minnesotensis* WT-3608, HD-29 and HOX-28 adhered to the cuticle of *H. glycines*. Bar = 10 μm (**D**) An increased in the relative expression of the *HIM-SKN7* gene was observed during the infection process. RNA was extracted from WT-3608, HD-29 and HOX-28 after 24 h of nematode inoculation. The actin gene was used as an internal control.

The ability of all strains to parasitize secondary stage juveniles (J2) of *H. glycines* was assayed on CMA in 12 wells tissue culture plates. First, a 10 μl conidia suspension (2 × 10^5^ conidia ml^−1^) of all strains was allowed to germinate for 14 days, and after which 100 J2 of SCN were inoculated into each well. The parasitized and alive nematodes were monitored at 48 h and 96 h under an inverted microscope. The ability of the knock out HD-29, to parasitize J2 was decreased by 20% as compared to WT-3608. For HOX-28, the parasitized J2 percentage was 100% at 96 h (Fig. 6A). However, when active and alive J2 with adhered conidia were calculated at 96 h, the percentage of alive nematodes was higher in the knockout strain, HD-29, while there was also a significant response between WT-3608 and the over-expressed HOX-28 to completely parasitize the nematodes (Fig. 6B,C). During the infection process, the relative expression of *HIM-SKN7* was increased 4- to 7-fold at 24 h of J2 inoculation in WT-3608 and HOX-28, respectively (Fig. 6D). The nematode parasitism assays provide an understanding into the role of *HIM-SKN7* in *H. minnesotensis* virulence and its efficiency in parasitizing the second stage juveniles of *H. glycines*.

## Discussion

Nematophagous fungi are natural enemies of plant pathogenic nematodes, and provide promising strategies for managing crops against nematodes in order to achieve high yields. The soybean cyst nematode is a ubiquitous plant parasitic nematode that is capable of causing huge losses in the soybean producing regions of the world, especially those that lack available management practices, including crop rotation and resistant cultivars^22^. *Hirsutella minnesotensis* is a competent bio-control agent of *H. glycines* that produces globose conidia that adhere to cuticle of secondary stage juvenile (J2) of SCN and eventually penetrate and digest the nematode. While the whole genome analysis of *H. minnesotensis* was previously published^2^, the specific functional genes that are expressed during stress, conidiation, parasitism and other cellular functions remain to be investigated. For this purpose, we characterized a highly conserved member of stress regulatory gene, *HIM-SKN7*, which shows high homology with the DNA binding domain of heat shock transcription factors.

Heat shock transcription factors (HSFs) play critical roles in regulating survival and normal metabolic processes under diverse stresses and acute conditions. The involvement of HSFs has been documented in conidiation, thermotolerance, virulence and the refolding of damaged proteins, and in particular, in maintaining cell homeostasis^8^,^23^,^24^. The SKN7 stress regulatory transcription factor was observed to interact with HSF1, and together, they resulted in the high induction of heat shock genes in response to oxidative stress in yeast and increased the sensitivity of yeast cells to oxidative agents in null mutants, which was associated with the regulation of different regulatory genes^14^,^16^,^17^. In the present study, *HIM-SKN7* was found to regulate various cellular functions, including conidiation, thermotolerance and resistance against abiotic stresses, apoptotic-like cell death and nematode parasitism in *H. minnesotensis*. Null mutant lacking *HIM-SKN7* showed reduced biomass formation and conidiation production, as compared to the wild type strain and *HIM-SKN7* over-expression mutant (Table 1). The regulation of some conidiation pathways by such conserved families of proteins in the bio-control fungus, *Coniothyrium minitans*, have been investigated and provide insights into genetically enhancing conidiation^8^,^25^,^26^. This indicates the regulation of conidiation in *H. minnesotensis* by *HIM-SKN7*, which is significant because plentiful conidia production by bio-control agents could lead to the manufacturing of commercial products for controlling plant pathogenic nematodes.

Thermotolerance and resistance against abiotic stresses are required by all organisms, including fungi, in order to survive in adverse environmental conditions. The heat shock response occurs via the induction of heat shock genes, and the disruption of such genes in different organisms has been shown to inhibit thermotolerance and increase the sensitivity of the organisms to abiotic stresses, including oxidative stress^16^,^27^,^28^,^29^. The over-expression of *HIM-SKN7* in *H. minnesotensis* increased its thermotolerance against heat and abiotic stresses, as compared to the wild type strain and knock out HD-29. This revealed that in this biocontrol agent fungus, SKN7 probably interacts with heat shock genes to induce thermotolerance. In fungi, *Drosophila melanogaster* and mammals, heat shock factor proteins have the fundamental capability for stress sensing and are converted from monomers to homotrimers in response to heat and oxidative stresses^8^,^28^,^30^,^31^. When the wild type strain was heat treated to 30 °C, no significant increases in the transcript level of *HIM-SKN7* was observed, as compared to control (25 °C), but an increase was observed at high temperatures. The heat treatments at 37 °C and 40 °C for 20 and 40 mins disturbed the normal growth of the wild type strain and knock out mutant, but the over-expression mutant successfully survived, and their mycelia and conidia were observed to be tolerant against heat and abiotic stresses. The increased expression of two HSFs (*HIM_00008* and *HIM_00329*) that play important roles in cellular functioning and thermotolerance was also observed (Fig. 4E), and the over-expression of these genes enhanced the regulation of tolerance to diverse environmental conditions and stresses^8^,^32^,^33^,^34^. These results emphasize the contribution of *HIM-SKN7* to stabilizing *H. minnesotensis* against heat and abiotic stresses by interacting with heat shock factor proteins.

The induction of apoptotic-like cell death by farnesol (FOH), a 15-carbon isoprenoid alcoholic natural compound and a main precursor of the isoprenoid or sterol biosynthesis pathway^35^, has been reported in many fungi, including *Metarhizium robertisii, Candida albicans, S. cerevisiae*, and *Aspergillus nidulans*^36^,^37^. In this study, we observed that FOH triggered cellular apoptosis, as well as necrosis like features, including intense chromatin condensation and marginalization, in the nematode endoparasitic fungus *H. minnesotensis*. The plate assay and microscopic observations showed low conidial germination at high FOH concentration (50 μM) in *HIM-SKN7* knock out strain, as compared to the wild type and over-expression strain. A recent study revealed that the deletion of Bax inhibitor 1 (Bl-1) perturbed fungal development, virulence and thermotolerance and also reduced fungal resistance to FOH, which suggests that cell death was mediated by the endoplasmic reticulum stress signaling pathway in entomopathogenic fungus *M. robertsii*^36^. These results revealed the role of *HIM-SKN7* in regulating cellular homeostasis against apoptosis-like cell death and providing tolerance against such alcoholic compounds.

Nematophagous fungi are a promising control strategy for plant pathogenic nematodes in the natural ecosystem. In the present study, we tested the ability of transformed strains to parasitize the secondary stage juveniles of *H. glycines. H. minnesotensis* is a cold-adapted fungus that preferentially parasitizes the cyst nematodes in nature^6^, and recently, a transformation system involving GFP-labeling was established in *H. minnesotensis* to investigate the infection process in *H. glycines*^3^. *H. minnesotensis* is abundant in soils with soybean monocultures, suggesting that it is an indicator of natural SCN suppressive soils in different locations in China and the USA^4^,^6^,^38^. The over-expression of *HIM-SKN7* enhanced conidial adherence and the parasitic capability of *H. minnesotensis*, as compared to the wild type strain (Fig. 6C). Moreover, the ability of the knock out HD-29 to parasitize the nematodes was reduced with that of WT-3608. At present, functional genetic studies in nematophagous fungi, especially in *Hirsutella* spp. are limited. Stress-related genes have been described to be involved in the process of virulence in insect pathogenic fungi^36^ and the nematode egg parasitic fungus *P. chlamydosporia*, and the induction of serine proteases and chitinase-encoding genes was previously investigated in the nematode-trapping fungus *Arthrobotrys oligospora*, during parasitism^39^,^40^. Our results showed the involvement of *HIM-SKN7* in nematode parasitism of *H. minnesotensis*.

In conclusion, this is the first study to report multi-functions of *HIM-SKN7* in nematode endoparasitic fungus *H. minnesotensis*. Our findings provide a comprehensive understanding of the role of this stress-responsive transcription factor in conidiation, thermotolerance, abiotic stresses, apoptotic-like cell death and virulence. The deletion of *HIM-SKN7* decreased the tolerance of the fungus towards acute temperature and abiotic stresses and reduced the endoparasitic ability of *H. minnesotensis* against *H. glycines*. Our results also indicate that *HIM_*SKN7 regulates apoptosis-like cell death and that its over-expression increased conidiation, which would facilitate the commercial production of this nematode endoparasitic fungus for controlling plant pathogenic nematodes.

## Materials and Methods

### Strains, media and cultural conditions

The *Hirsutella minnesotensis* wild type strain 3608 was maintained on PDA and used throughout this study. All the mutant strains were also maintained on potato dextrose agar (PDA) at 25 °C with appropriate antibiotics. The *Escherichia coli* strain DH5α, and the *Agrobacterium tumefaciens* strain EHA105 were prepared and stored at −80 °C. For cloning and plasmid DNA, bacterial strains were propagated in Luria Bertani (LB) medium supplemented with the appropriate antibiotic when required.

### Phylogenetic, gene structure and conserved motifs analyses

To establish the phylogenetic relationship between *HIM-SKN7* (KJZ76743.1) and its orthologs, protein sequences from representative fungal species belonging to different families in Phylum Ascomycota were aligned using the ClustalW program of the Molecular Evolutionary Genetics Analysis (MEGA version 6.0) software suite^41^. A maximum likelihood tree was generated with the WAG Model using the following parameters: a Nearest-Neighbor-Interchange heuristic method for tree inference, 1,000 bootstrap replications for phylogeny test, and a complete deletion for gaps/missing data. Gene structure, including introns and exons, was investigated using the online Gene Structure Display Server (http://gsds.cbi.pku.edu.cn/), which is based on genomic and coding sequences^42^. The conserved motifs were determined by the MEME online server^43^. The following parameters were used: maximum numbers of different motifs, 07; minimum motif width, 30; maximum motif width, 50; the other parameters were kept at their default settings. The tertiary structure of *HIM-SKN7* was constructed using SWISS-TOOL workshop^44^.

### Construction strategy of the *HIM-SKN7* disruption and over-expression vectors

To elucidate the function of *HIM-SKN7* gene, a gene disruption vector was constructed by following the homologous recombination strategy. The 968 bp and 987 bp DNA fragment from the upstream and downstream regions of *HIM-SKN7* were amplified from the wild type strain using the primers, 5Sac-FP/5Xma-RP and 3Asc-FP/3Pac-RP, respectively. Both PCR amplified fragments were sequenced to verify the true gene sequence. The purified fragments were cloned into the pMD18-T vector and digested with Sac1-Xma1 (upstream) and Asc1-Pac1 (downstream), before being ligated into the binary vector, pAg1-H3. Additionally, the disruption vector pAg1-H3 was digested with the same restriction enzymes to facilitate the ligation of the upstream and downstream fragments to the corresponding sites containing the hygromycin phosphotransferase (*hph*) gene cassette, resulting the formation of SKN7pAg1-H3.

To construct the over-expression vector, an over-expression cassette was created using promoter (PtrpC) and terminator (TtrpC) from *Aspergillus nidulans* with a 1.8 kb fragment of the *HIM-SKN7* ORF that was amplified from WT-3608 with the primer pair SKN7OX-F and SKN7OX-R. The hygromycin resistant gene cassette was constructed using the 1.4 kb *hph* gene and was ligated into PtrpC and TtrpC using the preliminary cloning vector, pSKH. The *HIM-SKN7* gene cassette and *hph* resistance gene cassette were ligated into the backbone of the pCAMBIA3300 binary vector by digesting it with Xcm1 and Xba1, respectively, resulting in the formation of SKN7ovx-V. To confirm the orientation of the *HIM-SKN7* gene in the over-expression vector, a forward primer from PtrpC and a gene specific reverse primer PromSP3/SKN7OX-R were used to amplified ~1.9 kb DNA fragment. The ligation of the *hph* gene cassette was confirmed by amplifying the 937 bp fragment with the primer pair Hyg-NF and Hyg-NR. All the primers are listed in Table 2.

### Transformation and screening of *HIM-SKN7* disruption and over-expression mutants

The disruption vector SKN7pAg1-H3 and the over-expression vector SKN7ovx-V were transformed into *A. tumefaciens* EHA105 using the heat shock method. Fresh conidia (10 days old) of *H. minnesotensis* were transformed with the *A. tumefaciens*-mediated transformation method, as previously described^8^. To screen for *HIM-SKN7* disrupted and over-expression transformants, candidates were grown on PDA supplemented with 200 μg/ml hygromycin B and 500 μg/ml cefotaxime. The positive transformants harboring the homologous recombination event were confirmed by PCR, RT-PCR and real-time RT-PCR.

### Expression profiling of HIM-SKN7 in Hirsutella minnesotensis

All strains were cultured for 4 days in PDB at 25 °C (150 rpm), and the collected mycelium was processed immediately for further RNA extraction. TRIzol reagent (Ambion) was used to extract RNA manually and treated with DNaseI (Invitrogen) by following the kit protocol. First strand cDNA synthesis was performed using FastQuant RT Kit (with gDNase) (TIANGEN) according to the manufacturer instructions.

Expression analysis was carried out by reverse transcriptase-PCR (RT-PCR) and SYBR green-based real-time RT-PCR on a CFX96 Real-Time System (Bio-Rad). A gene specific primer pair, HRT-F/HRT-R was used to amplify a 254 bp region of *HIM-SKN7* ORF by RT-PCR. The reaction condition were 3 min at 95 °C and, 30 cycles of 40 s at 94 °C, 30 s at 51 °C and 30 s at 72 °C, followed by 10 min at 72 °C. The real-time RT-PCR conditions were 2 min at 95 °C and, 40 cycles of 20 s at 95 °C, 15 s at 51 °C and 20 s at 72 °C. The *HIM-Actin* gene was amplified with the primer pair, Actin-F/Actin-R, and was used as an internal control for all reactions.

### Heat shock and abiotic stress assay

To test the thermotolerance of all strains under different temperatures, temperature ranges for heat shock assay were initially determined by culturing the WT-3608 on PDA plates, and incubating the plates at 18 °C, 25 °C, 30 °C, 37 °C and 40 °C for 2 weeks. After the initial screening, mycelium from *H. minnesotensis* WT-3608, knock out HD-29 and over-expressed HOX-28 were subjected to heat shock treatment at 37 °C for 2 h to determine the incubation time that was required. Finally, the heat shock treatment for conidia of all strains was performed at 37 °C and 40 °C for 20 and 40 minutes in a water bath. The heat-treated conidial suspensions (1 × 10^5^ conidia ml^−1^; 10 μl) were inoculated in PDB and incubated for 3 days at 25 °C (150 rpm) for the examination of conidia germination and survival. To test the transcriptional activity of the gene, *HIM-SKN7*, during the heat stress, its relative expression levels were measured by real-time RT-PCR.

All strains were cultured on PDA supplemented with 5 mM H_2_O_2_, 1 M mannitol and 2% ethanol for 14 days. Radial hyphal growth was measured to observe the tolerance of all strains to stress conditions. For the conidia survival assay, conidia were collected in 0.05% Tween-80, and spore suspensions (1 × 10^5^ conidia ml^−1^; 10 μl) from all strains were inoculated in PDB amended with 5 mM H_2_O_2_, 1 M mannitol and 2% ethanol. The samples were incubated for 3 days at 25 °C (150 rpm) to record germination and survival percentages. The relative expression of *HIM-SKN7* was measured for each treatment. All strains were generated in triplicate, and the experiment was repeated twice.

### Apoptosis assay

The 10-day old germlings of WT-3608, knock out HD-29 and over-expressed HOX-28 were treated with the apoptosis-inducing compound, farnesol (FOH, TCI), at concentrations of 15 μM and 50 μM along with control for 4 h at 25 °C. The dyes Hoechst 33342 and PI (Shanghai Yeasen Biotechnology Co. Ltd.) were used to stain nuclei and necrotic cells, respectively. Observations were made using fluorescence microscopy with a ZEISS microscope (Axio Imager.A2). The spore germination percentage of all strains was determined by conidia treatment with 50 μM FOH for 24 h, and conidial suspensions (1 × 10^5^ conidia ml^−1^; 10 μl) were transferred to PDB for 3 days and on PDA for 14 days at 25 °C. All strains were generated in triplicate, and the experiment was repeated three times.

### Nematode parasitism bioassays

To reveal the role of the *HIM-SKN7* in the parasitism of *H. glycines*, a 10 μl conidial suspension (2 × 10^5^ conidia ml^−1^) of all strains were thoroughly spread onto 1 ml corn meal agar in 12 well tissue culture plates (Nest Biotechnology Co. Ltd.). The plates were incubated at 20 °C for 14 days and then, 100 second-stage juveniles (J2) of *H. glycines* were placed in 20 μl of sterilized water suspension and inoculated into each well. The plates were placed in an illuminated flow hood to allow the evaporation of excess water from each well in order to enhance the adhesion of conidia with the J2 of *H. glycines*. Nematodes were washed out from the wells of the plate with 1 ml of 0.05% Tween-80 at 48 h and 96 h. Using an inverted microscope (CK40-F200, Olympus), the J2 with conidia attached to cuticle and parasitized nematodes were calculated based on the total number of nematodes collected using an inverted microscope (CK40-F200, Olympus). The mycelia of WT-3608, HD-29 and HOX-28 were collected at 24 h after nematode inoculation and the relative expressions of *HIM-SKN7* were measured. All strains were generated in triplicate, and the experiment was repeated three times.

### Statistical analysis

Analysis of variance (ANOVA) was used for evaluating the results. Duncan’s Multiple Range (DMR) test was used to compare the treatment means values. Treatments were considered significant when *P* ≤ 0.05.

## Additional Information

**How to cite this article**: Hussain, M. *et al*. The transcription factor *SKN7* regulates conidiation, thermotolerance, apoptotic-like cell death and parasitism in the nematode endoparasitic fungus *Hirsutella minnesotensis. Sci. Rep.* **6**, 30047; doi: 10.1038/srep30047 (2016).

## Figures and Tables

**Table 1:** Comparison of phenotypic characteristics between the mutants and wild type 3608 of *H. minnesotensis*.

Strains	Colony diameter^A^(mm)	Biomass^B^(mg/plate)	Conidiation^C^ (10^6^conidia/plate)
WT-3608	14.5 ± 0.34^b^	22.50 ± 0.95^b^	2.41 ± 0.14^b^
HD-29	14.83 ± 0.31^b^	21.83 ± 0.91^b^	1.29 ± 0.11^c^
HOX-28	16.33 ± 0.21^a^	59.67 ± 2.21^a^	3.28 ± 0.23^a^

(**A**) Colony diameter was recorded on PDA plates following incubation at 25 °C for 14 days. (**B**) Biomass (fresh-weight) was calculated from 14-day-old cultures. (**C**) Conidia production in14-day-old cultures were counted using a haemocytometer. The mean values and standard error were calculated from 6 replicates. The letters within each column indicate statistically significant differences of *P* ≤ 0.05.

**Table 2:** The primers used in this study.

Primer	Sequence (5’-3’)	Expected Size	Roles	Source
**Primer used for clone DNA from** ***H. minnesotensis***				
5Sac-FP	CGAGCTCCTGGTAAAATCTGTCCCG	968 bp	Amplified 5′ UTR and partial sequence of *HIM-SKN7*	This study
5Xma-RP	CCCCGGGCTGTCGGATAAAACTGGA			This study
3Asc-FP	GGCGCGCCAATAAGGTGATGAAGCAA	987 bp	Amplified 3′ UTR and partial sequence of *HIM-SKN7*	This study
3Pac-RP	CTTAATTAAACCCAATGCGGCACAAGC			This study
SKN7OX-F	TCTCAAGGTAGCGGCAACAAT	1.8 kb	Amplified *HIM-SKN7* ORF for over-expression and to confirm replacement with *hph* cassete	This study
SKN7OX-R	CCCGCACTGTCTCCATCGTGT			This study
**Primer used for identification of null mutants**				
5-HD-FP	GCCGAGGACTTCACCACTA	741 bp	Amplified *HIM-SKN7* gene (PCR 1)	This study
3-HD-RP	GCAGCCCATTGACTTGAGA			This study
HD-F5N	GGCAGTTCGTGGTTTAGTCG	2.2 kb	Amplified sequence of UTR and partial sequence of *hph* gene (PCR 2)	This study
Hyg-R3	CTTTGTAGAAACCATCGGCG			This study
Hyg-NF	AAGTTCGACAGCGTCTCC	937 bp	Amplified the gene *hph* (PCR 3)	This study
Hyg-NR	TTCCACTATCGGCGAGTA			This study
**Primer used for identification of over-expression mutants**				
PromSP3	AGGTAAGTGAACGACCCGGTC	1891 bp	Amplifying ORF and partial sequence of PtrpC (PCR4)	This study
SKN7OX-R	CCCGCACTGTCTCCATCGTGT			This study
Hyg-NF	AAGTTCGACAGCGTCTCC	937 bp	Amplified the gene *hph* (PCR 5)	This study
Hyg-NR	TTCCACTATCGGCGAGTA			This study
**Primer used for RT-PCR and RT-qPCT**				
HRT-F	ACCTAAGCCCATACAACG	254 bp	mRNA transcription of *HIM-SKN7* gene	This study
HRT-R	TAAATCATTCATCGGGTCAT			
HIM-08F	GGAAACCCAGAAACGACCCT	149 bp	mRNA transcription of *HIM_00008* gene	This study
HIM-08R	AGTCGGGTTCGGCTTGTTGT			
HIM-29F	TTCTGTATGGGCTGCGTCTC	181 bp	mRNA transcription of *HIM_00329* gene	This study
HIM-29R	TCTGGCTCATCAACAAACCC			
Actin-F	CCCCATCTACGAGGGTTT	554 bp	Used as internal control	This study
Actin-R	GGGAAGCGAGAATGGAAC			
